# Assessing the knowledge of key one health elements among African higher education students: African multi- center cross-sectional study

**DOI:** 10.1186/s12889-025-22935-6

**Published:** 2025-05-19

**Authors:** Fatma A. Amer, Nkalubo Jonathan, Hana A. Nofal, Rehab Tash, Noha M. Hammad, Manar G. Gebriel, Elichilia Robert Shao, Ibrahim Eldaghayes, Djamila Meriane, Ayman A Allam, Heba Mohtady Ali, Raghda A. Hafez, Mohamed Elahmady, Hend E.S. Khalil, Maysaa A. Saeed, Shaker Wagih Shaltout, Siaka Samake, Alassane Dicko, Connie Walyaro, Gohole Akaranga Arthur, Bashir Jarrar, Almahamoudou Mahamar

**Affiliations:** 1 Department of Medical Microbiology and Immunology, Faculty of Medicine, Zagazig University, Zagazig, Egypt; 2 Mulago National Referral Hospital, Kampala, Uganda; 3 Department of Public Health and Community Medicine, Faculty of Medicine, Zagazig University, Zagazig, Egypt; 4 Department of Internal Medicine, KCMC University, Moshi, Tanzania; 5 Department of Microbiology and Parasitology, Faculty of Veterinary Medicine, University of Tripoli, Tripoli, Libya; 6 Department of Plant Biology and Ecology, Faculty of Natural and Life Science, Ferhat Abbas Setif 1 University, Setif, Algeria; 7 Griffith University, Gold Coast, Australia; 8 Microbiology Laboratory, Al Ahli Hospital, Al Doha, Qatar; 9 Microbiology and Immunology Laboratory, Armed Forces Hospital, Al Doha, Qatar; 10 Department of Tropical Medicine, Faculty of Medicine, Zagazig University, Zagazig, Egypt; 11 Department of Tropical Medicine, Faculty of Medicine, Portsaid University, Portsaid, Egypt; 12 University of Sciences, Techniques, and Technologies of Bamako, Bamako, Mali; 13 International Society for Infectious Diseases/Talk AB[M]R, Nairobi, Kenya; 14 Kenya Medical Research Institute, Nairobi, Kenya; 15 Climate Change Unit, College of Sciences, Jerash University, Jerash, Jordan

**Keywords:** Africa, AMR, Climate change, One health, Zoonosis

## Abstract

**Background:**

The One Health (OH) approach addresses the interconnectedness of human, animal, and environmental health, playing a critical role in tackling antimicrobial resistance (AMR), zoonotic diseases, and climate change. Despite its importance, limited research has examined OH knowledge among African students - future professionals in public health, veterinary science, and environmental fields.

**Objectives:**

This study assesses OH knowledge and its key components - AMR, zoonosis, and climate change - among higher education students across 26 African countries.

**Methods:**

A cross-sectional, multicentre study was conducted from June 2023 to February 2024, using an online survey distributed in English and French. The survey targeted higher education students from diverse academic disciplines. OH knowledge levels were evaluated based on median scores, and statistical analysis using Statistical Package for the Social Sciences (SPSS) software version 24 identified regional and disciplinary variations.

**Results:**

A total of 726 students from 26 African Countries participated in the study. 88.2% of Central African students demonstrated adequate OH knowledge, while students from North Africa exhibited the lowest scores (64.1%). Non-medical students outperformed medical students in OH awareness (64.1% vs. 45.2%, *p* < 0.001). The most important OH issues recognized by participants included OH major goal (86.6%), concept approach (83.5%), shared health threats by people, animal, and environment (82.1%). However, knowledge gaps were evident in awareness about Carbapenem-resistant Enterobacterales (65.3%), animal as an early warning sign of human illness (65%), zoonosis related to environmental toxicants. Key knowledge gaps were identified in awareness about Carbapenem-resistant Enterobacterales (65.3%), animal as an early warning sign of human illness (65%), zoonosis related to environmental toxicants. However, no specific knowledge gaps were identified related to impact of climate change on health.

**Conclusion:**

While OH awareness among African students is relatively high, significant regional disparities and knowledge gaps remain, particularly in AMR and zoonotic disease prevention. Strengthening interdisciplinary education, enhancing regional OH initiatives, and incorporating OH into university curricula are crucial for fostering a well-informed future workforce.

**Supplementary Information:**

The online version contains supplementary material available at 10.1186/s12889-025-22935-6.

## Background

The one health (OH) approach has emerged as a critical paradigm in the quest to tackle complex global health challenges. Rooted in the understanding that human health, animal health, and environmental health are intrinsically linked, OH promotes interdisciplinary collaboration and integrated solutions [1]. This approach is particularly relevant for addressing pressing issues such as antimicrobial resistance (AMR), zoonotic diseases, and the impacts of climate change [2, 3].

AMR is a critical element of the OH framework. It is an escalating crisis accelerated by the misuse and overuse of antimicrobials in human medicine, veterinary practices, and agriculture. In Africa, the challenge of AMR is compounded by a lack of regulatory enforcement, and inadequate surveillance systems [4]. Africa has the world’s highest mortality rate from AMR infections, resulting in over 27 deaths per 100,000 [5].

Zoonotic diseases are a persistent threat in Africa, where many countries are considered ‘hot spots’ for emerging and reemerging infectious diseases [6]. In Africa, a 63% jump in diseases spread from animals to people has been seen in the last decade. Nipah virus, human immunodeficiency virus (HIV), Ebola, Hantavirus, and Lyme disease are examples [7]. Emergent pathogens might have the capacity for pandemic potential among humans, as observed in the current Coronavirus disease 2019 (COVID-19) global emergency [8]. There is over $3.6 trillion loss from COVID-19, $53 billion loss from the 2014–2016 Ebola outbreak, $20 billion for Zika and $8.6 billion for canine rabies [9]. To combat zoonotic diseases, the African Union establishes the OH Coordination Group on Zoonotic Diseases [9].

Climate change is increasingly recognized as a significant determinant of health, creating both direct and indirect risks to global health, particularly within vulnerable healthcare systems [10]. Changing temperature and precipitation patterns can alter the distribution of vector-borne diseases such as malaria and dengue fever [11]. Extreme weather events can disrupt food systems, aggravate malnutrition, and lead to human displacement with subsequent strain on the already suboptimal healthcare systems. In Africa, the impacts of climate change are particularly severe, intensifying existing vulnerabilities and creating new health challenges [12].

The rise of these common dangers to public health can be prevented and countered with the OH strategies. The primary step in developing resilient and adaptive OH strategies is the existence of relevant knowledge among those actually and/or potentially involved in implementation.

Despite the growing recognition of OH in global health policies, there is limited research on OH knowledge among African students, who are the future professionals in health, veterinary, and environmental sciences. Understanding their knowledge levels is crucial for shaping educational and policy interventions [13]. Moreover, few studies have assessed OH awareness among African students, and existing research has not comprehensively examined regional disparities or the influence of different academic disciplines on OH knowledge [14, 15]. Given the increasing importance of OH in addressing global health threats, it is essential to assess the awareness and understanding of this approach among future professionals. Hence, this study aims to fill this gap by assessing OH knowledge across different academic disciplines in Africa. Furthermore, findings from this study can help universities incorporate OH concepts into curricula, promote interdisciplinary learning, and guide policymakers in strengthening OH initiatives across Africa.

## Methods

### Study design

This study adopted a cross-sectional multicenter design involving African higher education students.

### Study participants

The study included participants from African countries located in the 5 main regions of Africa, as aligned with the United Nations’ geoscheme; North Africa and the sub-Saharan area which is further divided into; Western sub-Saharan Africa, Central sub-Saharan Africa, Eastern sub-Saharan Africa, and Southern sub-Saharan Africa [16]. Any participant studying in an African country and from non-African country was excluded from the study.

### Sample size

A minimum sample size of 384 was calculated using Open Epi info according to the following input: 55.6% satisfactory scores about one health at 95% CI and 5 degrees of freedom [17].

### The survey tool

The survey tool was developed in English and French. A specialized bilingual expert back-translated the French version to ensure accuracy.

The questionnaire was developed following a structured process to assess students’ knowledge about OH. First, the goals were identified based on key competencies in disciplines as informed by the literature. The necessary information was then determined by reviewing relevant studies and existing assessment tools to ensure comprehensive coverage of essential knowledge and skills [17, 18]. Questions were crafted in alignment with these goals, adhering to research-based guidelines to ensure clarity, minimize bias, and capture a broad range of knowledge [19]. An expert panel of ten specialists from each discipline was engaged to evaluate content validity. The panel consisted of faculty members, PhD-qualified academics, and educational experts. They assessed the tool’s items for relevance and appropriateness. This interdisciplinary team ensured that the final product was academically rigorous, relevant, and accessible to students [20]. A reliability test was done using the reliability coefficients, which was high and suitable for scientific purposes (Cronbach’s alpha ranged from 0.78 to 0.90).

A final questionnaire was generated based on the feedback comments and revised accordingly. A pilot was conducted on 10% of the sample who were not included in the results. The questionnaire was then finalized with the required modifications, mainly the length of the questions, clarity and simplicity.

The questionnaire, composed of 70 questions, was organized into 5 sections; section one: to collect the demographic data about the participants (6 questions), then 4 sections; (1) OH general knowledge, (2) Knowledge about AMR, (3) Zoonosis- related information, and (4) Climate change awareness. The OH general knowledge section includes 21 questions, the AMR section comprises 11 questions, the zoonosis section consists of 15 questions, and the climate change section is made of 17 questions (Suppl. 1). For all questions assessing knowledge, correct answers received a score of 1. Incorrect answers and do not know were assigned a 0 [21].

The scores were summed to yield a total score of OH ranging from 0 to 21, for the second section; scores were summed to yield a total score from 0 to 11. Total score of zoonosis ranged from 0 to 15, and total score of climate change knowledge ranged from 0 to 16. Total knowledge score included summation of four sections to yield a total score ranging from 0 to 63. A higher score indicated adequate knowledge when the cut-off is at the median of observation [21, 22].

The questionnaire was shared between 26 African countries, and data was gathered from June 2023 to February 2024 using a Google form, which was distributed both directly via emails and social media channels, and by snowball process. Snowball is a sampling technique in which existing study participants assist researchers in recruiting other potential subjects [23].

### Statistical analysis

Data were analyzed using the Statistical Package for the Social Sciences (SPSS) software version 24. Categorical variables were displayed as numbers and percentages (n %). Qualitative data were analyzed using Chi-square test and Fischer exact test when appropriate. To identify independent factors correlated with adequate knowledge of OH, categorical variables were converted to dummy variables. A probability (p) value ≤ 0.05 was considered significant at 95% CI.

## Results

A total of 26 African country answered the questionnaire. Data were collected from 726 higher education students from North Africa; Egypt: 308- Libya: 20-Tunisiaia: 15- Algeria: 14, West sub-Saharan; Mali 91- Nigeria 26- Senegal 21, Central sub- Saharan; Cameroon 16- Democratic Republic of the Congo (DRC) 18- East sub-Saharan; Kenya 37- Rwanda 15- South Sudan 30- Tanzania 41- Uganda 28- Zimbabwe 15, South sub-Saharan; Batswana 16. Due to the very small number of student participants, ten countries were excluded from the statistical analysis. These countries are Burkina Faso, Côte d’Ivoire, Madagascar, Malawi, Sierra Leone, Somaliland, South Africa (1 participant), Seychelles, Somalia (2 participants), and Ethiopia (4 participants).

Students were categorized into (1) Health disciplines—medicine, dentistry, pharmacy, veterinary medicine, and nursing, (2) Non-Health disciplines, represented by agriculture and interdisciplinary disciplines, including fields that provide foundational training in some African countries as an introduction to the OH, such as environmental health, animal production, conservation biology, and global health.

### Sociodemographic characteristics of participant students

Students aged 24 years old and older exhibited a significant level of knowledge (*p* < 0.001). However, 55.1% of females significantly displayed adequate level of knowledge compared to 43.6% of males (*p* = 0.002). Residence had no significant impact on total knowledge. The total knowledge was significantly more adequately observed within non-medical students (64.1%) compared to medical ones. Overall, among the 5 main regions of Africa, 88.2% of participants from Central Africa demonstrated a significant level of knowledge (*p* < 0.001). However, the lowest level of knowledge was prevalent among students from North Africa (64.1%). The relations between level of knowledge of the study populations and age, sex, residence, education specialty, and African region are demonstrated in Table 1.

**Table 1:** Relation between demographic characteristics and total knowledge of participants (*N* = 711)

Variable	Inadequate Knowledge *n* = 365 *n* (%)	Adequate Knowledge *n* = 346 *n* (%)	*P*- value
Age < 24 (*n* = 362) ≥ 24 (*n* = 349)	230 (63.5) 135 (38.7)	132 (36.5) 214 (61.3)	< 0.001*
**Sex** Male (*n* = 399) Female (*n* = 312)	225 (56.4) 140 (44.9)	174 (43.6) 172 (55.1)	0.002*
**Residence** Rural (*n* = 223) Urban (*n* = 488)	124 (55.6) 241 (49.4)	99 (44.4) 247 (50.6)	0.12
**Educational discipline** Medical (*n* = 580) Non-medical (*n* = 131)	318 (54.8) 47 (35.9)	262 (45.2) 84 (64.1)	< 0.001*
**African region** North Africa (*n* = 357) Western Africa (*n* = 138) Central Africa (*n* = 34) Eastern Africa (*n* = 166) Southern Africa (*n* = 16)	229 (64.1) 69 (50.0) 4 (11.8) 55 (33.1) 8 (50.0)	128 (35.9) 69 (50.0) 30 (88.2) 111 (66.9) 8 (50.0)	< 0.001*

*Significant difference

### Analysis of OH related general knowledge

**Fig. 1:**
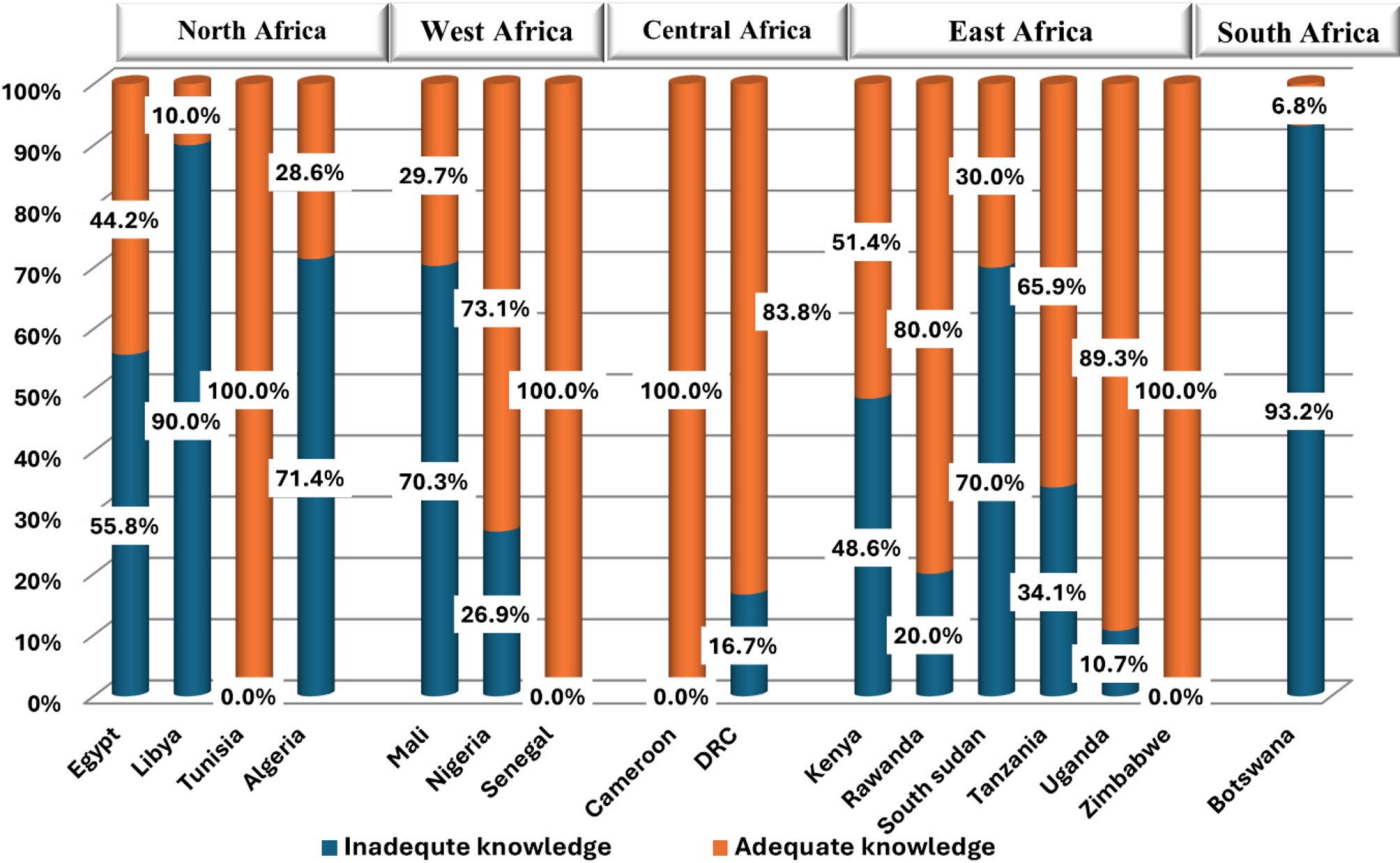
Bar graph demonstrating the level of overall knowledge about one health among students from participant African countries

When comparing the extent of OH general knowledge among students from regions, 88.2% of participants from the Central Sub-Saharan region were superior (compared to others (*p* < 0.001). Students from Egypt (55.8%), Libya (90%), Algeria (71.4%), Mali (70.3%), South Sudan (70%) and Botswana (93.2%) had the lowest levels of OH general knowledge (Fig. 1).

Notable knowledge gaps were encountered as regards country’ membership of any of OH project or network (51.3%). In addition, more than half of students were unaware if their country is involved in OH initiatives (surveillance, prevention and control of zoonosis, environmental contaminant in foods, antimicrobial resistance) or their institutions endorsed /adopted OH (59.1%, and 58.2%, respectively) (Table 2).

**Table 2:** Knowledge of participants about one health (*N* = 711)

Question	Yes (%)	No (%)	Don’t know (%)
1. Did any of your curricula refer to one health?	466 (65.5)	122 (17.2)	123 (17.3)
2. Is one health concept collaborative, interdisciplinary, inter-sectoral multi-institutional approach linking many different forms of knowledge and expertise?	594 (83.5)	19 (2.7)	98 (13.8)
3. Is the major goal of the one health approach to achieve optimal health outcomes recognizing interconnection between people, animals, plants and their shared environment?	616 (86.6)	14 (2.0)	81 (11.4)
4. Is your country member of any of OH project or networks?	346 (48.7)	43 (6.0)	322 (45.3)
5. Do common one health issues include zoonotic diseases?	555 (78.1)	29 (4.1)	127 (17.9)
6. Do common one health issues include antimicrobial resistance?	582 (81.9)	23 (3.2)	106 (14.9)
7. Do common one health issues include food safety and food security?	570 (80.2)	36 (5.1)	105 (14.8)
8. Do common one health issues include environmental contamination?	581 (81.7)	33 (4.6)	97 (13.6)
9. Do common one health issues include other health threats shared by people, animals, and environment?	584 (82.1)	25 (3.5)	102 (14.3)
10. Are you currently involved in OH initiatives (surveillance, prevention and control of zoonosis, environmental contamination in foods, antimicrobial resistance)?	291 (40.9)	341 (48.0)	79 (11.1)
11. Are medical practitioners and veterinarians only involved in one health activities?	152 (21.4)	416 (58.5)	143 (20.1)
12. Has your institution endorsed /adopted one health?	297 (41.8)	113 (15.9)	301 (42.3)
13. How relevant are the following one health advantages described in literature approach? 13.1 Early detection of threat and timely effective and rapid response 13.2 More effective disease control and/or biosecurity measures 13.3 Economic benefit and increase economic efficiency 13.4 Improvement in human or animal health (well-being) 13.5 Higher quantity of information and improved knowledge and skills 13.6 Ecosystem benefit 13.7 Personal or social benefits 13.8 Design of health policies	492 (69.2) 518 (72.9) 453 (63.7) 520 (73.1) 493 (69.3) 477 (67.1) 442 (62.2) 499 (70.2)	25 (3.5) 43 (6.0) 63 (8.9) 48 (6.8) 35 (4.9) 38 (5.3) 72 (10.1) 34 (4.8)	194 (27.3) 150 (21.1) 195 (27.4) 143 (20.1) 183 (25.7) 196 (27.6) 197 (27.7) 178 (25.0)
14. Are there in your country recent initiatives to encourage intersectoral collaboration (at administrative level) aimed to global advocacy of one health approach?	399 (56.1)	64 (9.0)	248 (34.9)

### Analysis of antimicrobial resistance related knowledge

**Fig. 2:**
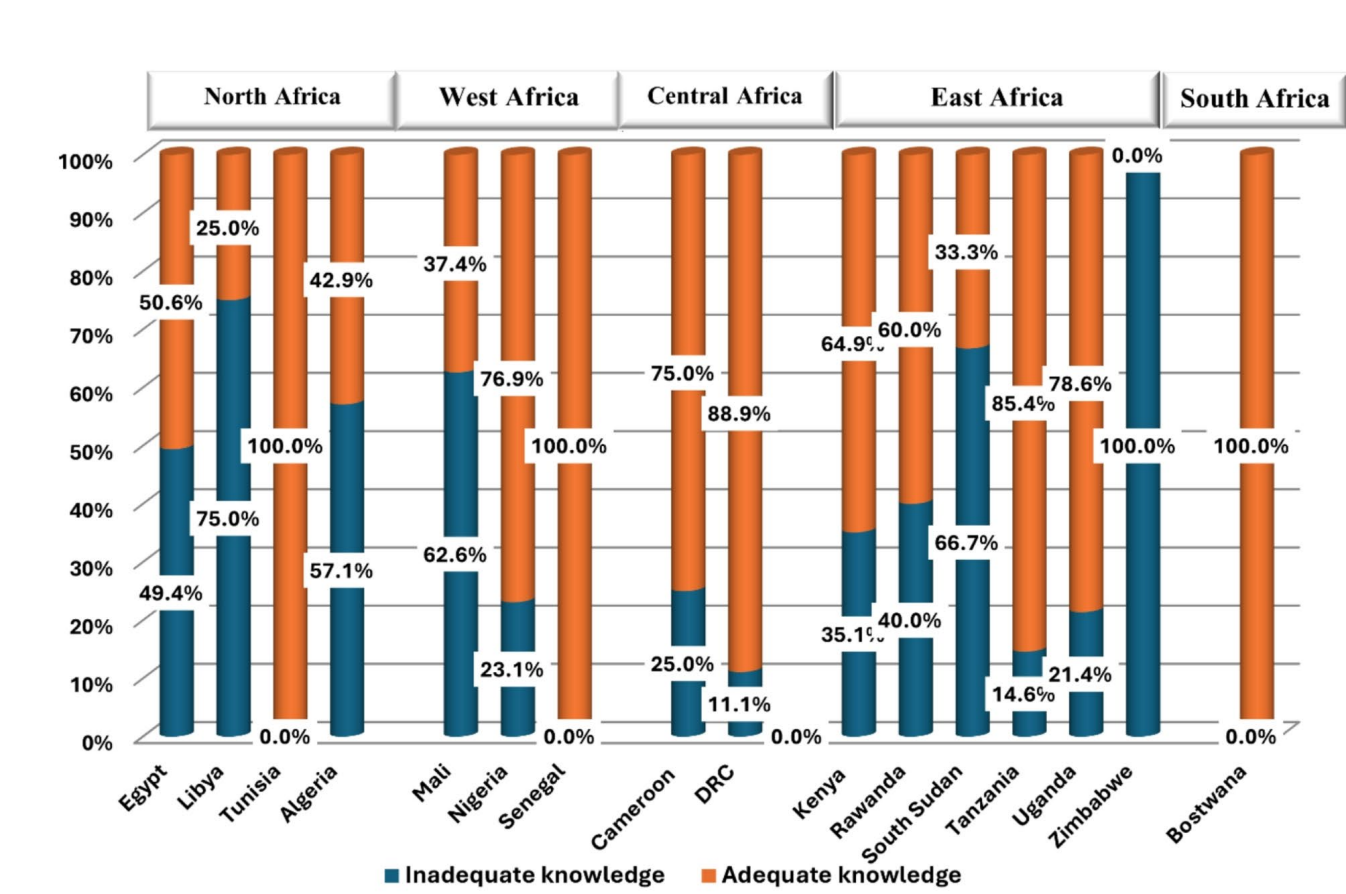
Bar graph demonstrating the level of knowledge of antimicrobial resistance among students from participant African countries

The optimum levels of knowledge regarding AMR were observed among students from Tunisia (100%), Senegal (100), and Botswana (100%), while those from Libya (75%), Algeria (57.1%), Mali (62.6%), South Sudan (66.7%), and Zimbabwe (100%) showed the lowest levels of AMR awareness (Fig. 2). Knowledge gap was evident concerning the superbug Carbapenem-resistant Enterobacterales (CRE) in 65.3% of participant students (Table 3).

**Table 3:** Knowledge of participants about antimicrobial resistance (*N* = 711)

Question	Yes (%)	No (%)	Don’t know (%)
1. Does your country Contribute to the AMR (antimicrobial resistance) monitoring with specific program?	425 (59.8)	71 (10.0)	215 (30.2)
2. Is there an ongoing antibiotic abuse in therapeutics in veterinary sectors?	411 (57.8)	52 (7.3)	248 (34.9)
3. Do you know about the critically important list of antimicrobial specified by the WHO?	365 (51.3)	216 (30.4)	130 (18.3)
4. Is antibiotic resistance a serious public health issue?	612 (86.1)	33 (4.6)	66 (9.3)
5. Is antibiotic resistance natural as well as anthropogenic (caused by humans or their activities)?	543 (76.4)	63 (8.9)	105 (14.8)
6. Does irrational antibiotics use in animals lead to resistance in humans?	463 (65.1)	87 (12.2)	161 (22.6)
7. Are you familiar with superbug CRE?	247 (34.7)	304 (42.8)	160 (22.5)
8. Are you familiar with livestock associated methicillin resistance staphylococcus aureus (LA-MRSA)?	401 (56.4)	206 (29.0)	104 (14.6)
9. Does the use of expired antibiotics lead to emergence of resistance?	435 (61.2)	88 (12.4)	188 (26.4)
10. Does injudicious use of antibiotics lead to antibiotic residues in milk and meat?	504 (70.8)	53 (7.5)	154 (21.7)
11. Does antibiotic residues in milk/meat lead to emergence of resistance?	481 (67.6)	44 (6.2)	186 (26.2)

### Analysis of zoonosis related knowledge

**Fig. 3:**
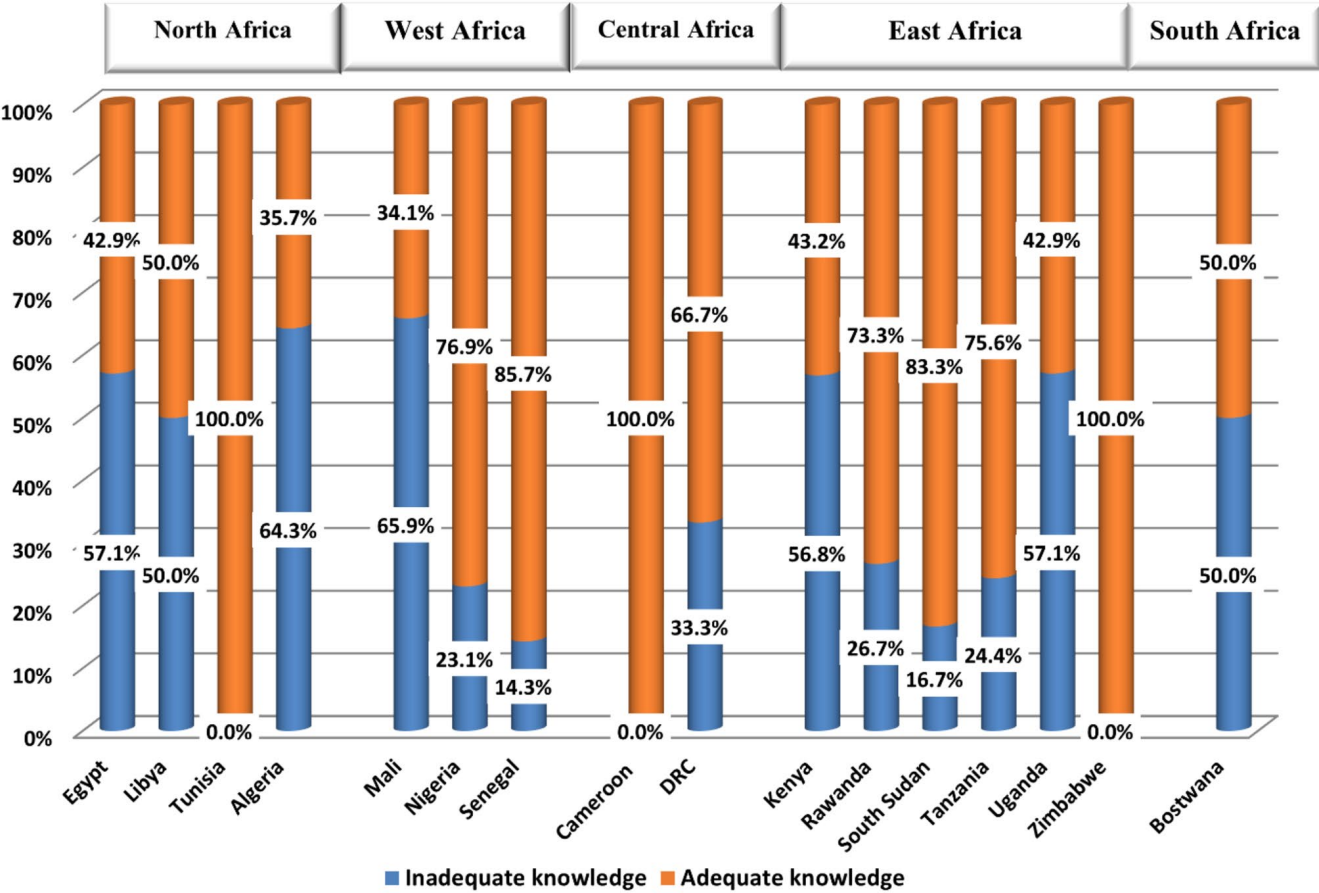
Bar graph demonstrating the level of knowledge about zoonosis among students from participant African countries

While all students from Tunisia, Cameron, and Zimbabwe demonstrated highest levels of knowledge about zoonosis, those from Egypt (57.1%), Algeria (64.3%), Mali (65.9%), Kenya (56.8%), and Uganda (57.1%) exhibited the lowest levels. In Libya and Botswana, the levels of good and poor understanding of zoonosis were evenly split. However, the students from the remaining countries displayed acceptable levels of knowledge (Fig. 3).

65% of the study participants could not know that animal can serve as an early warning sign of human illness. In 55.7% of students, suboptimal knowledge related to awareness about zoonosis caused by exposure to environmental toxicants was evident. Moreover, knowledge gaps were identified in understanding specific zoonoses (brucellosis and leptospirosis), their modes of transmission, and whether these diseases affect humans, animals, or both (Table 4).

**Table 4:** Knowledge of participants about zoonosis (*N* = 711)

Question	Yes *n* (%)	No *n* (%)	Don’t know *n* (%)
1.Can animals serve as early warning signs of potential human illness?	249 (35.0)	357 (50.2)	105 (14.8)
2.Have you received any training on zoonosis?	311 (43.7)	375 (50.2)	43 (6.0)
3.Do you know any diseases that people can catch from livestock?	540 (75.9)	85 (12.0)	86 (12.1)
4.Do you know any disease that people can catch from rodents?	489 (68.8)	119 (16.7)	103 (14.5)
5.Do you know any disease that people can catch from dogs?	585 (82.3)	66 (9.3)	60 (8.4)
6.Do you know any disease that can cause abortion in livestock?	377 (53.0)	219 (30.8)	115 (16.2)
7.Have you heard about a disease called brucellosis?	545 (76.6)	120 (16.9)	46 (6.5)
8.Have you heard about a disease called leptospirosis?	423 (59.5)	214 (30.1)	74 (10.4)
9.Have you heard about a disease called typhoid fever?	609 (85.7)	68 (9.6)	34 (4.8)
10.Do you know the ways that people can become infected with brucellosis?	423 (59.5)	194 (27.3)	94 (13.2)
11. Do you know the ways that people can become infected with Leptospirosis?	210 (29.5)	307 (43.2)	194 (27.3)
12.Do you know the ways that animals can become infected with brucellosis?	303 (42.6)	264 (37.1)	144 (20.3)
13.Do you know the ways that animals can become infected with leptospirosis?	210 (29.5)	307 (43.2)	194 (27.3)
14.Is there existing and active cooperation between ministry of health and ministry responsible for veterinary medicine when dealing with zoonosis in your country?	391 (55.0)	86 (12.1)	234 (32.9)
15.Are you aware of zoonosis caused by exposure to environmental toxicants?	315 (44.3)	219 (30.8)	177 (24.9)

### Analysis of climate change related knowledge

**Fig. 4:**
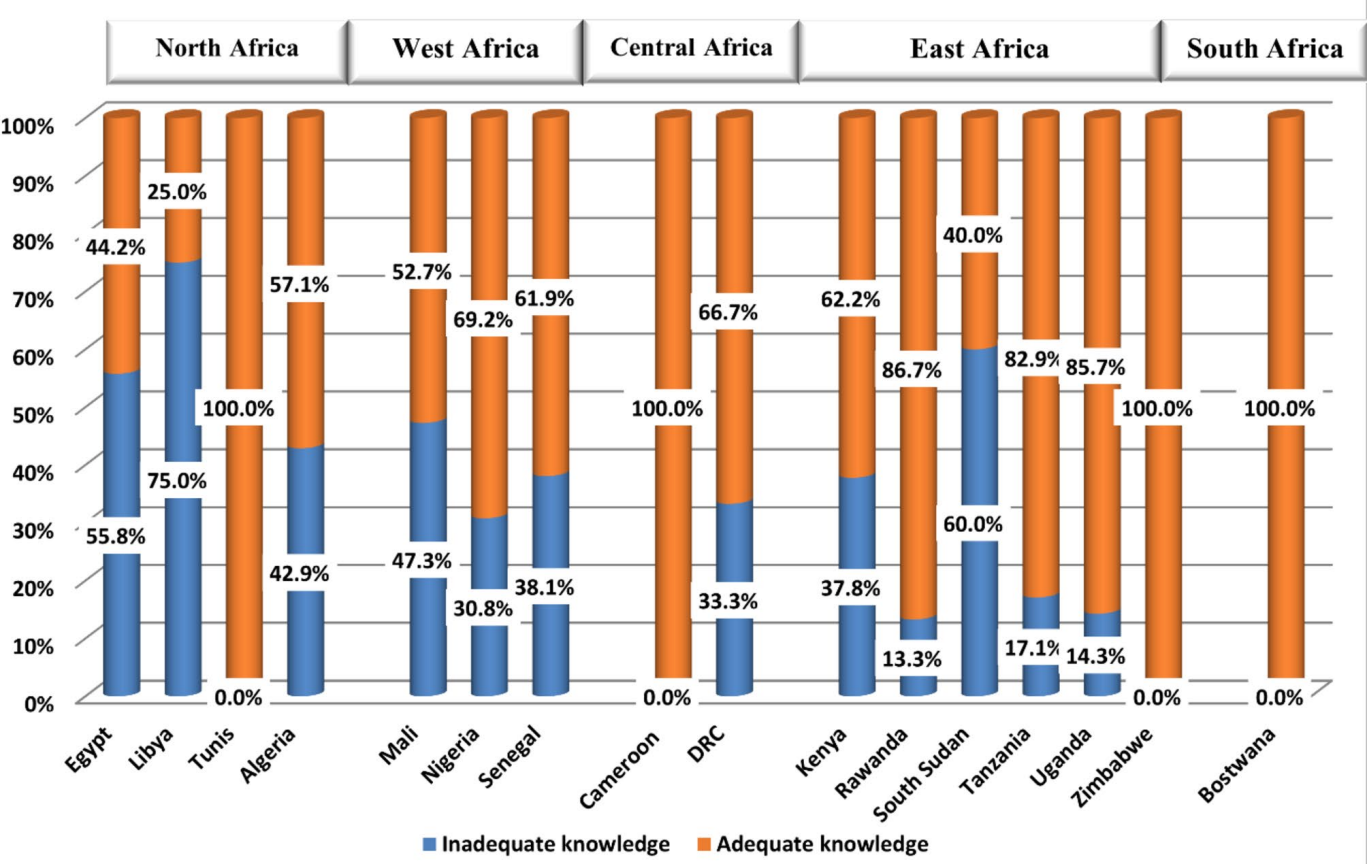
Bar graph demonstrating the level of knowledge about climate change impact on health among students from participant African countries

**Fig. 5:**
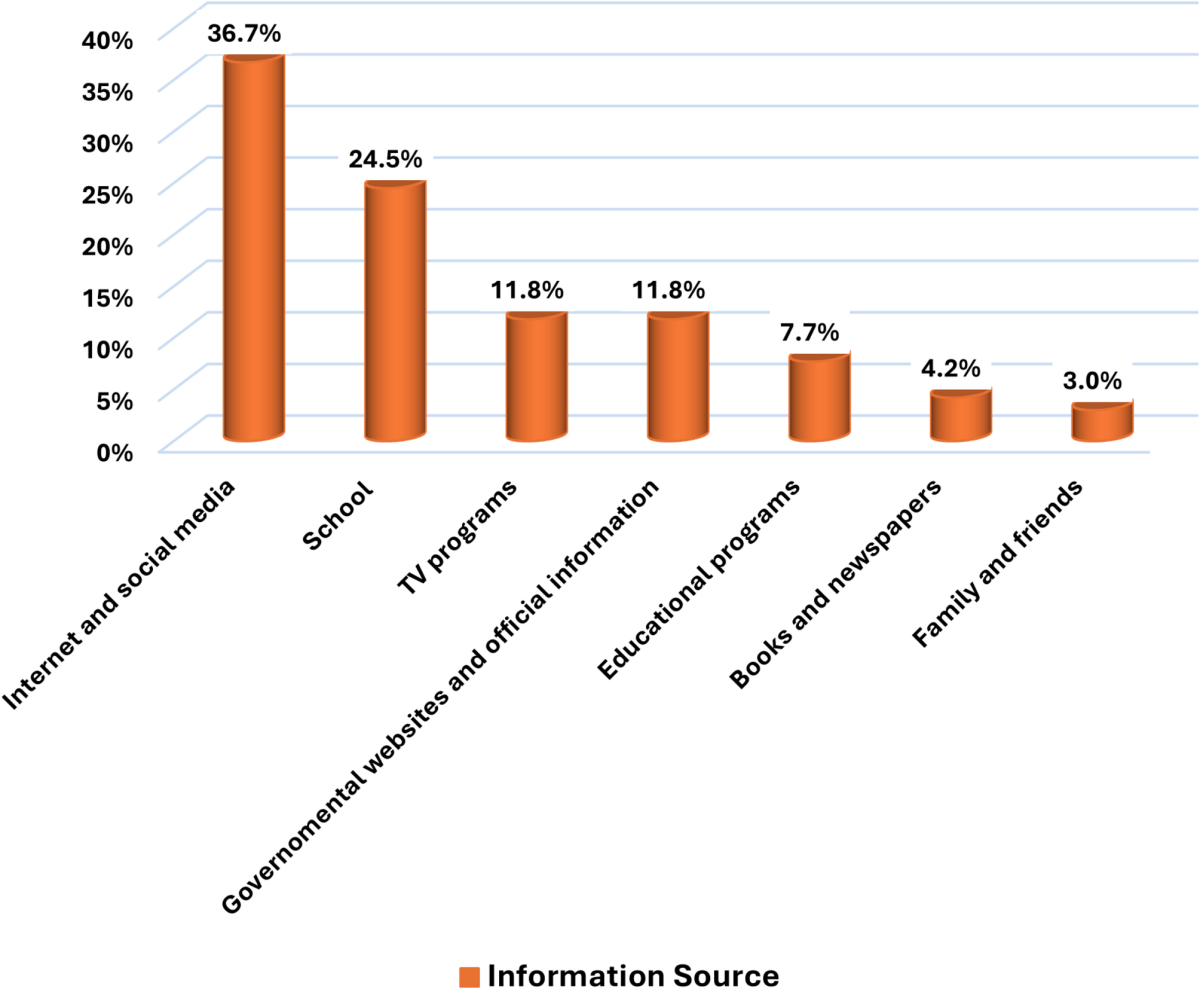
Bar graph showing the most important information source about climate change and global warming among participant students

All students from Tunisia, Cameron, Zimbabwe, and Botswana demonstrated optimal knowledge as regards the impact of climate change on health (100%). Although, students from Egypt (55.8%), Libya (75%), and South Sudan (60%) had the lowest level of knowledge (Fig. 4), no specific knowledge gap was identified (Table 5). Eventually, when students were asked about the most important source form which they get their information about global warming and climate change, internet and social media ranked first among their preferences (36.7%) (Fig. 5).

**Table 5:** Knowledge of participants about climate change (*N* = 711)

Question	Yes (%)	No (%)	Don’t know (%)
1.Have you heard the term “global warming”	635 (89.3)	36 (5.1)	40 (5.6)
2.Does global warming have an impact on human health?	630 (88.6)	27 (3.8)	54 (7.6)
3.Dose climate change increase the incidence of floods?	579 (81.4)	42 (5.9)	90 (12.7)
4.Does climate change increase the water shortage problem?	575 (80.9)	51 (7.2)	85 (12.0)
5.Does climate change increase the rate of glaciers melting?	578 (81.3)	35 (4.9)	98 (13.8)
6.Does climate change increase the possibility of extreme heat waves?	591 (83.1)	36 (5.1)	84 (11.8)
7.Does climate change increase the possibility of extreme cold?	500 (70.3)	80 (11.3)	131 (18.4)
8.Does climate change increase the spread of diseases that are transmitted from person to another such as gastroenteritis?	432 (60.8)	102 (14.3)	144 (24.9)
9.Does climate change increase the prevalence of malnutrition diseases?	508 (71.4)	63 (8.9)	140 (19.7)
10.Does climate change increase the likelihood of non –communicable diseases such as lung diseases, asthma and respiratory problems?	527 (74.1)	68 (9.6)	116 (16.3)
11.Does climate change affect mental health, increase anxiety and depression?	504 (70.9)	58 (8.2)	149 (21.0)
12.Can climate change impede health institutions to perform their role during severe cold spells or extreme heat?	521 (73.3)	61 (8.6)	129 (18.1)
13.Does climate change displace people and increase the number of refugees?	557 (78.3)	50 (7.0)	104 (14.6)
14.Do developed countries contribute more to climate change?	525 (73.8)	53 (7.5)	133 (18.7)
15.Are developing countries more vulnerable to the effect of climate change?	536 (75.4)	58 (8.2)	117 (16.5)
16.Will climate change be more severe in the future?	574 (80.7)	27 (3.8)	110 (15.5)

## Discussion

The OH approach which addresses health threats at the human-animal-environment interface is applicable at the subnational, national, regional, and global levels [24]. This study evaluated OH knowledge, and three of its key elements AMR, zoonotic diseases, and climate change, among students in 16 African countries to identify awareness and educational gaps.

Older students demonstrated greater knowledge than younger counterparts, likely due to prolonged education, diverse life experiences, and more developed cognitive maturity [25]. Females were more knowledgeable than males, perhaps due to their tendency to pursue health-related careers, stronger communication skills, and proactive health information-seeking behavior. Women often engage in collaborative learning, and exhibit interdisciplinary interests aligning with OH [26, 27].

Non-medical students were found to be more knowledgeable than medical students. Unlike medical curricula focused on clinical skills, non-medical programs often cover broader issues like climate change and encourage interdisciplinary collaboration and practical learning opportunities [28].

The overall knowledge of OH and its critical elements are higher among students in sub-Saharan African countries than in North African countries, except for Tunisia which has made significant progress in adopting the OH approach. Through various initiatives and policies, Tunisia is improving health outcomes by aligning strategies across sectors and is enhancing its ability to tackle complex health challenges such as zoonotic diseases, promoting a healthier, more sustainable future for all [29]. It is to be emphasized that all participants from Tunisia were veterinarians. Veterinarians are vital to the OH approach, their training equips them to recognize how environmental factors, animal health, and human health are intertwined, allowing them to contribute valuable expertise in collaborative efforts to promote health and prevent disease outbreaks [30].

The success of sub-Saharan countries over North Africa in OH awareness can be attributed to the extensive efforts and initiatives implemented in sub-Saharan regions to promote education and understanding of OH principles. In fact, there are 293 OH initiatives spread across 46 sub-Saharan countries [31]. These initiatives promote cross-sector collaboration to address OH key elements. Principal networks in the region include One Health Central and Eastern Africa (OHCEA) [32], the Southern African Centre for Infectious Disease Surveillance (SACIDS) [33], the Africa One Health University Network (AFROHUN) [34] the EcoHealth Alliance [35], and the Africa CDC One Health Program [36].

Despite the higher knowledge levels in sub-Saharan Africa, variations exist between regions and individual countries within the same regions. This may be attributed to differences in study methodologies, sample sizes, or the degree of OH implementation. The current study has classified sub-Saharan countries into three categories based on the relationship between OH initiatives, efforts, and levels of knowledge. The first category is characterized by good initiatives and efforts that successfully promote awareness. It includes Cameroon, DRC, Tanzania, Rwanda, Nigeria, and Senegal where substantial OH initiatives, supported by international collaborations and targeted programs [29, 37], have successfully enhanced knowledge among students. The second category comprises countries where significant initiatives and efforts have been made, yet the response remains suboptimal. South Sudan [38], Mali [39] Kenya [40], and Uganda [41] exemplify this situation. The limited impact of these initiatives reveals a gap in communication and implementation, highlighting the need for more effective educational campaigns and improved knowledge dissemination strategies. Strengthening messaging, expanding outreach, and ongoing evaluation are essential for achieving greater and lasting impact. In South Sudan [32], the planned implementation of the National Action Plan on Antimicrobial Resistance (AMR) and in Kenya [33], the recent rollout of the One Health Strategic Plan for Zoonotic Diseases (2021–2025) offer hope for future improvements. The third category includes regions without formal initiatives or efforts yet still showcasing a considerable level of knowledge. Botswana serves as an example [42]. The participants’ strong understanding of AMR may be due to Botswana’s National Action Plan on AMR (2021–2022) [35]. In contrast, their solid knowledge of climate change seems to be influenced by informal efforts or other external sources of information. Similarly, Zimbabwe, although lacking a national OH coordinating body [43], awareness of OH issues is notably high. In October 2024, Zimbabwe took a significant step towards adopting a OH strategic plan [44]. This plan will guide the country’s efforts to combat zoonotic diseases, antimicrobial resistance (AMR), and other emerging health threats, while also enhancing the overall health system.

The key knowledge gaps in OH activities highlighted by this survey, which is limited awareness of the roles of higher authorities in OH efforts in Egypt, Libya, Algeria, and some sub-Saharan countries, are likely due to factors like limited media coverage, ineffective communication, and public mistrust. In Libya, political instability worsens this issue. Bridging these gaps requires media enhancement, transparency, and public engagement. The second gap relating to CRE, which can impact appropriate diagnosis and poor infection control, should be addressed by educational campaigns and better surveillance systems.

Despite general awareness of zoonotic diseases, knowledge of transmission routes is lacking, needing further training. Nevertheless, the relatively high awareness of climate change among respondents, except in Libya and Egypt, may be attributed to the numerous initiatives hosted in Africa which are supported by international partners and local organizations, e.g., Africa Climate Mobility Initiative [45] coupled by involving youth in awareness raising activities [46]. Furthermore, traditional African knowledge plays a role in fostering climate awareness. The South African student’s demonstration of strong knowledge could be due to unique and robust local initiatives in this area [47].

In this study, the primary sources of OH information for students included the internet, social media, TV programs and educational programs. Recognizing the power of these platforms, many organizations use social media, blogs, mobile apps, and other digital tools to spread credible information [48]. However, it seems that communication gaps persist in conveying complex information effectively and reaching a wider audience. Addressing this gap will require strategic efforts in media relations, transparency, and community engagement.

The One Health High-Level Expert Panel (OHHLEP), established in May 2021, has adopted a theory of change (ToC) which drove the OH form a theoretical concept to a practical multisectoral collaborative work at different levels [49, 50].

The ToC can be leveraged to bridge the knowledge gaps and improve OH awareness and application in African countries by aligning educational initiatives with system changes. The pathways of change include integration of OH concepts into the national policy of higher education in African countries, emphasis on the need of institutional support to policy application, advocation of OH principles’ integration, particularly into medical, veterinary, and environmental science programs, foundation of regional centers of excellence in OH education [15]. In context, the importance of integrating OH concepts into educational curricula has been acknowledged by countries like Kenya, Uganda, Tanzania, Ethiopia, Rwanda, Nigeria, South Africa, Zambia, Ghana, and Cameroon [1, 15].

Moreover, capacity building for educators and researchers by fellowships, training program, awareness campaigns and fund support of conducting research and field OH application can be implicated. These educational and training programs can focus on limiting prevalence of AMR, identifying drivers of zoonotic spillover and zoonotic disease surveillance, and adapting strategies for climate change [51].

In addition, building up networking between students via encouraging engagement to OH initiatives and university clubs, establishing Pan-African OH student networks to encourage students to exchange knowledge, share experiences, participate in conference and collaborate on projects [34, 50]. Furthermore, social media and digital platforms can facilitate student networking and collaboration, provide real-time accessible information, publish OH- related updates, and present infographics and videos for quick learning and comprehension.

By implementing these strategies, African higher education institutions and policymakers can strengthen OH awareness, foster cross-sector collaboration, and equip the next generation of professionals with the knowledge necessary to tackle global health challenges.

## Conclusion

In conclusion, while there are commendable efforts and improvements in OH awareness across Africa, significant knowledge gaps remain. Addressing these gaps requires continued investment in OH education, improved interdisciplinary collaboration, and targeted regional initiatives to ensure comprehensive understanding and effective implementation of OH strategies. Moreover, if the awareness in some African countries (Egypt, Libya, Algeria, and Mali) of the concept one health and its key components is deficient, our study rings the alarm bell that this awareness is even not satisfactory in countries (Mali and South Sudan) that are rich in all initiatives and capabilities, which requires changing future plans later on.

### Limitations of the study

Proportional allocation could not be applied in sample size calculation for this multicenter study because the target population comprised higher education students rather than the general population, and data on the number of higher education students in each African country were unavailable. Moreover, we acknowledge the potential selection bias because the questionnaire was designed to circulate within African countries directly or via snowball distribution using emails, social media, and direct messages, albeit 26 African countries were accessible and answered the questionnaire with at least one response per country. Accordingly, countries with less than 15 responses were excluded from the statistical analysis. Although some regions were underrepresented due to logistical and political challenges in reaching the necessary target participants, the current study included a relatively satisfactory sample size from 16 African countries representing Africa. The non-response bias caused by limited participation from these regions was not due to a lack of interest but rather the inability to establish channels of contact with the targeted survey respondents. Overcoming these communication barriers will be essential in future studies to ensure broader participation and more comprehensive data collection across all regions. However, as one of the first multi-center studies in Africa to include 26 countries, this marks an unprecedented achievement in previous research and may inspire future participation from underrepresented or non-represented African countries.”

## Electronic supplementary material

Below is the link to the electronic supplementary material.

Supplementary Material 1

## Data Availability

The datasets analysed during the current study are available from the corresponding author on reasonable request.

## Abbreviations

ACDI: African Climate & Development Initiatives
AFROHUN: Africa One Health University Network
AMR: Antimicrobial Resistance
AYICC: African Youth Initiative on Climate Change
CCAO: Climate Change Africa Opportunities
CDC: Centers for Disease Prevention and Control
CRE: Carbapenem Resistant Enterobacterales
DRC: Democratic Republic of Congo
OH: One Health
OHCEA: One Health Central and Eastern Africa
SACIDS: Southern African Centre for Infectious Disease Surveillance
SAYCCC: South African Youth Climate Change Coalition
VS: Veterinary Services
