# Supplementary material for: Assessing the knowledge of key one health elements among African higher education students: African multi- center cross-sectional study
Source: BMC Public Health. 2025 May 19;25:1846. doi: 10.1186/s12889-025-22935-6 (PMC12087165; doi:10.1186/s12889-025-22935-6)
Supplement: Supplementary file 1 — Supplementary Material 1 [file 12889_2025_22935_MOESM1_ESM.pdf]

Assessing the Knowledge of One Health: African Multi-Center Study

The aim of this study is to assess the knowledge of one health and its Key Elements: Antimicrobial Resistance, Zoonosis, and Climate Change
Among African Higher Education Students

* Indicates required question

Declarations

1. **I understand the purpose of the study and all information taken from this study will be coded to protect each subject's name. No names or other identifying information will be used when discussing or reporting data.** *

   Check all that apply.

   ☐ Yes, I understand

2. **By proceeding to next sections, I voluntarily agree to participate in this study** *

   Check all that apply.

   ☐ Yes, I agree

Sociodemographic data

3. **Q1. Initials** *

   ____

4. **Q2. Country** *

Dropdown

Mark only one oval.

- Algeria
- Angola
- Benin
- Botswana
- Burkina Faso
- Burundi
- Cameroon
- Cape Verde
- Central African Republic
- Chad
- Comoros
- Congo
- Cote d'Ivoire
- Democratic Republic of Congo
- Djibouti
- Egypt
- Equatorial Guinea
- Eritrea
- Eswatini (Swaziland)
- Ethiopia
- Gabon
- Gambia
- Ghana
- Guinea
- Guinea-Bissau
- Kenya
- Lesotho
- Liberia
- Libya
- Madagascar
- Malawi
- Mali
- Mauritania
- Mauritius
- Morocco
- Mozambique
- Namibia
- Niger
- Nigeria
- Rwanda
- São Tomé and Príncipe
- Senegal
- Seychelles
- Sierra Leone
- Somalia
- South Africa
- South Sudan

Sudan
United Republic of Tanzania
Togo
Tunisia
Uganda
Zambia
Zimbabwe

5. **Q3. Residence** *

Mark only one oval.

Rural
Urban

6. **Q4. Age** *

____

7. **Q5. Sex** *

Mark only one oval.

Male
Female

8. **Q6. What is your education specialty?** * Dropdown

Mark only one oval.

Medicine
Dentistry
Pharmacy
Veterinary
Agriculture
Nursing
Physiotherapy
Other

Knowledge about one health

9. *
Q1. Did any of your curricula refer to the One Health?

Mark only one oval.

Yes
No
Don't know

10. **Q2. Is one health concept collaborative, interdisciplinary, inter-sectoral multi-institutional approach linking many different forms of knowledge and expertise?**

Mark only one oval.

- Yes
- No
- Don't know

11. **Q3. Is the major goal of the one health approach to achieve optimal health outcomes recognizing interconnection between people, animals, plants and their shared environment?** *

Mark only one oval.

- Yes
- No
- Don't know

12. **Q4. Is your country a member of any of one health projects or networks?** *

Mark only one oval.

- Yes
- No
- Don't know

13. **Q5. Do common one issues health include zoonotic diseases?** *

Mark only one oval.

- Yes
- No
- Don't know

14. **Q6. Do common one health issues include antimicrobial resistance?** *

Mark only one oval.

- Yes
- No
- Don't know

15. **Q7. Do common one health issues include food safety and food security?** *

Mark only one oval.

- Yes
- No
- Don't know

16. **Q8. Do common one health issues include environmental contamination?** *

Mark only one oval.

- Yes
- No
- Don't know

17. **Q9. Do common one health issues include other health threats shared by people, animals, and environment?** *

Mark only one oval.

- Yes
- No
- Don't know

18. **Q10. Are you currently involved in one health initiatives (surveillance, prevention and control of zoonosis, environmental contamination in foods, antimicrobial resistance)?** *

Mark only one oval.

- Yes
- No
- Don't know

19. **Q 11. Are medical practitioners and veterinarians only involved in one health activities?** *

Mark only one oval.

- Yes
- No
- Don't know

20. **Q12. Has your institution endorsed /adopted one health?** *

Mark only one oval.

- Yes
- No
- Don't know

21. **Q13. How relevant are the following one health advantages described in the literature approach? ***

Mark only one oval per row.

	Yes	No	Don't know
13.1 Early detection of threat and timely, effective or rapid response	⬭	⬭	⬭
13.2 More effective disease control and /or biosecurity measures	⬭	⬭	⬭
13.3 Economic benefit and increase economic efficiency	⬭	⬭	⬭
13.4 Improvement in human or animal health (well-being)	⬭	⬭	⬭
13.5 Higher quantity of information and improved knowledge and skills	⬭	⬭	⬭
13.6 Ecosystem benefit	⬭	⬭	⬭
13.7 Personal or social benefits	⬭	⬭	⬭
13.8 Design of health policies	⬭	⬭	⬭

22. **Q.14 Are there in your country recent initiatives to encourage intersectoral collaboration (at administrative level) aimed to global advocacy of one health approach? ***

Mark only one oval.

- ⬭ Yes
- ⬭ No
- ⬭ Don't know

knowledge about antimicrobial resistance

23. **Q1. Does your country contribute to antimicrobial resistance monitoring with specific programs?**

Mark only one oval.

- Yes
- No
- Don't know

24. **Q2. Is there an ongoing antibiotic abuse in therapeutics in veterinary sectors?** *

Mark only one oval.

- Yes
- No
- Don't know

25. **Q3. Do you know about the critically important list of antimicrobials specified by the WHO?** *

Mark only one oval.

- Yes
- No
- Don't know

26. **Q4. Is antibiotic resistance a serious public health issue?** *

Mark only one oval.

- Yes
- No
- Don't know

27. **Q5. Is antibiotic resistance natural as well as anthropogenic (caused by humans or their activities)?** *

Mark only one oval.

- Yes
- No
- Don't know

28. **Q6. Does irrational antibiotics use in animals lead to resistance in humans?** *

Mark only one oval.

- Yes
- No
- Don't know

29. **Q7. Are you familiar with superbug carbapenem resistant Enterobctericea (CRE)?** *

Mark only one oval.

- Yes
- No
- Don't know

30. **Q8. Are you familiar with livestock associated methicillin resistance staphylococcus aureus (LA-MRSA)?** *

Mark only one oval.

- Yes
- No
- Don't know

31. **Q9. Does the use of expired antibiotics lead to emergence of resistance?** *

Mark only one oval.

- Yes
- No
- Don't know

32. **Q10. Does injudicious use of antibiotics lead to antibiotic residues in milk and meat?** *

Mark only one oval.

- Yes
- No
- Don't know

33. **Q11. Does antibiotic residues in milk/ meat lead to emergence of resistance?** *

Mark only one oval.

- Yes
- No
- Don't know

Knowledge about Zoonosis

34. **Q1. Animal cannot serve as early warning signs of potential human illness, can they?** *

Mark only one oval.

- Yes
- No
- Don't know

35. **Q2 Have you received any training on zoonosis?** *

Mark only one oval.

- Yes
- No
- Don’t know

36. **Q3. Do you know any diseases that people can catch from livestock?** *

Mark only one oval.

- Yes
- No
- Don’t know

37. **Q4. Do you know any disease that people can catch from rodents?** *

Mark only one oval.

- Yes
- No
- Don’t know

38. **Q5. Do you know any disease that people can catch from dogs?** *

Mark only one oval.

- Yes
- No
- Don’t know

39. **Q6. Do you know any disease that can cause abortion in livestock?** *

Mark only one oval.

- Yes
- No
- Don’t know

40. **Q7.Have you heard about a disease called brucellosis?** *

Mark only one oval.

- Yes
- No
- Don’t know

41. **Q8. Have you heard about a disease called leptospirosis?** *

Mark only one oval.

- Yes
- No
- Don’t know

42. **Q9. Have you heard about a disease called typhoid fever?** *

Mark only one oval.

- Yes
- No
- Don’t know

43. **Q10. Do you know the ways that people can become infected with brucellosis?** *

Mark only one oval.

- Yes
- No
- Don’t know

44. **Q11. Do you know the ways that people can become infected with Leptospirosis?** *

Mark only one oval.

- Yes
- No
- Don’t know

45. **Q12. Do you know the ways that animals can become infected with brucellosis?** *

Mark only one oval.

- Yes
- No
- Don’t know

46. **Q13. Do you know the ways that animals can become infected with leptospirosis?** *

Mark only one oval.

- Yes
- No
- Don’t know

47. **Q14. Is there existing and active cooperation between ministry of health and ministry responsible for veterinary medicine when dealing with zoonosis in your country?** *

Mark only one oval.

- Yes
- No
- Don't know

48. **Q15. Are you aware of zoonosis caused by exposure to environmental toxicants?** *

Mark only one oval.

- Yes/Oui
- No/Non
- Don't know /ne sais pas

Knowledge about climate change

49. **Q1. Have you heard the term "global warming"** *

Mark only one oval.

- Yes
- No
- Don't know

50. **Q2.Does global warming have an impact on human health?** *

Mark only one oval.

- Yes
- No
- Don't know

51. **Q3.Dose climate change increase the incidence of floods?** *

Mark only one oval.

- Yes
- No
- Don't know

52. **Q4. Does climate change increase the water shortage problem?** *

Mark only one oval.

- Yes
- No
- Don't know

53. **Q5. Does climate change increase the rate of glaciers melting?** *

Mark only one oval.

- Yes
- No
- Don’t know

54. **Q6. Does climate change increase the possibility of extreme heat waves?**

Mark only one oval.

- Yes
- No
- Don’t know

55. **Q7. Does climate change increase the possibility of extreme cold?** *

Mark only one oval.

- Yes
- No
- Don’t know

56. **Q8. Does climate change increase the spread of diseases that are transmitted from person to another such as gastroenteritis?** *

Mark only one oval.

- Yes
- No
- Don’t know

57. **Q9. Does climate change increase the prevalence of malnutrition diseases?** *

Mark only one oval.

- Yes
- No
- Don’t know

58. **Q10. Does climate change increase the likelihood of non –communicable diseases such as lung diseases, asthma and respiratory problems?** *

Mark only one oval.

- Yes
- No
- Don’t know

59. **Q11. Does climate change affect mental health, increase anxiety and depression?** *

Mark only one oval.

- Yes
- No
- Don’t know

60. **Q12. Can climate change impede health institutions to perform their role during severe cold spells or extreme heat?** *

Mark only one oval.

- Yes
- No
- Don’t know

61. **Q13. Does climate change displace people and increase the number of refugees?** *

Mark only one oval.

- Yes/Oui
- No/Non
- Don’t know /ne sais pas

62. **Q14. Do developed countries contribute more to climate change?** *

Mark only one oval.

- Yes
- No
- Don’t know

63. **Q15. Are developing countries more vulnerable to the effect of climate change?** *

Mark only one oval.

- Yes
- No
- Don’t know

64. **Q16. Will climate change be more severe in the future?** *

Mark only one oval.

- Yes
- No
- Don’t know

65. **Q17. What are the most important sources you get your information about global warming and climate change from? You can choose more than one answer.** *

Check all that apply.

- [ ] Books and newspapers
- [ ] Governmental websites and official information
- [ ] Educational programs
- [ ] Family and friends
- [ ] Internet and social media
- [ ] Tv programs
- [ ] School

This content is neither created nor endorsed by Google.

Google Forms
